# Content analysis of nutritional information in paediatric oral health education leaflets

**DOI:** 10.1186/s12887-017-0814-z

**Published:** 2017-02-20

**Authors:** Amit Arora, Jenny Doan, Jessamine Martinez, Colin Phan, Gregory S. Kolt, Sameer Bhole, Mark Fort Harris, Jane Anne Scott, Debra Hector

**Affiliations:** 1 School of Science and Health, Western Sydney University, Campbelltown, NSW Australia; 2 Discipline of Paediatrics and Child Health, Sydney Medical School, Westmead, NSW Australia; 3 Oral Health Service, Sydney Local Health District and Sydney Dental Hospital, NSW Health, Surry Hills, NSW Australia; 4 COHORTE Research Group, Ingham Institute of Applied Medical Research, Liverpool, NSW Australia; 5 Faculty of Dentistry, The University of Sydney, Westmead, NSW Australia; 6 Centre for Primary Health Care and Equity, UNSW Australia, Randwick, NSW Australia; 7 School of Public Health, Curtin University, Perth, WA Australia; 8 Cancer Australia, Surry Hills, NSW Australia

**Keywords:** Content analysis, Children, Nutrition, Oral health, Leaflets, Health education

## Abstract

**Background:**

The aim of this study was to determine if paediatric oral health education leaflets with a food and nutritional focus provide messages that are clear and consistent with the current Australian Dietary Guidelines and the Infant Feeding Guidelines.

**Methods:**

Forty-three leaflets aimed at parents were sourced from Australian state and territory Health Departments, oral health industry partners and commercial organisations, and a content analysis was performed. Recommendations on food and drink type, consumption frequency and general diet and nutrition advice were considered and cross-referenced with the Australian Dietary Guidelines and the Infant Feeding Guidelines to identify areas of consistency and discrepancy.

**Results:**

Twenty leaflets recommended reducing the consumption of sugary and/or acidic food, while 23 leaflets recommended reducing the consumption of sugary and/or acidic drinks. The majority of the leaflets advised water (n = 35) and milk (n = 23) to drink. Although 33 leaflets encouraged a healthy diet, seven of these did not specify what a healthy diet was. Twenty-eight leaflets provided early childhood-related (0–2 years) feeding advice. Confusing messages were found in nine leaflets, with ambiguous recommendations that were open to individual interpretation.

**Conclusions:**

There were some inconsistencies between the leaflets and the dietary and infant feeding guidelines in Australia; and across the leaflets, as not all important messages were included in any one leaflet. Government Health Departments and other relevant agencies should ensure that advisory messages regarding diet, particularly those with dental implications, are clear, complete and consistent across all dental educational leaflets.

## Background

Dental caries (decay) is a preventable oral disease affecting children, teenagers, and adults worldwide [1]. Despite the marked decline in dental caries in developed countries over the past 30 years, the prevalence of dental caries remains unacceptably high in children and is a major public health problem [2]. Bagramian et al. [3] reported that epidemiological data from different countries show that there is a marked increase in the prevalence of dental caries since the mid-1990s on a global scale. In 2007, it was noted that 46% of Australian children aged 6 years had an average of two ‘decayed teeth’, ‘teeth missing due to caries’, or ‘filled primary (baby) teeth’ [4]. Armfield and Spencer [5] have reported that since the mid-1990s, the caries experience in Australian children has increased in primary teeth and the burden appears to be carried over to permanent (adult) teeth.

Although dental caries has a multifactorial aetiology, dietary sugars play a significant role in caries initiation and development [6, 7]. In recent years there has been a call for oral health promotion to utilise the common risk factor approach [8, 9] as diets high in sugar have been linked to both obesity and dental caries [7, 10]. Although both of these chronic conditions are preventable, their prevalence in young children has been on the rise since mid-1990s [11] raising concern over the consumption of foods high in sugar and fat [12].

Free sugars, which include added sugars and sugars naturally present in honey, syrups and fruit juices are cariogenic [7]. The WHO recommends that both adults and children should reduce the intake of free sugars to less than 10% of total energy intake [7], however the results of the 2011–2012 Australian Health Survey identified that 52.2% of children aged 2–3 years old and 68.5% children aged 4–8 years exceeded this recommendation [13]. There is overwhelming evidence to suggest the cariogenic potential of different children’s food and snack items [2, 14, 15]. In particular, items such as confectionery, cookies/biscuits, cake, sugar-sweetened beverages, and dried fruit are considered to be highly cariogenic [2, 14, 15]. On the other hand, fresh fruit is considered to be low in cariogenicity and items such as nuts, vegetables, and cheese and other dairy products are considered to be non-cariogenic [2, 14, 15]. In light of this evidence, the key dental-related dietary messages in the 2013 Australian Dietary Guidelines [16] and the 2012 Infant Feeding Guidelines [17] are:

1. Infants should be exclusively breastfed until around 6 months of age followed by the introduction of solid foods and breastfeeding should be continued until 12 months of age, and beyond if desired;
2. If the infant is not breast-fed, infant formulas should be used as an alternative to breast milk until 12 months of age and putting the infant to bed with a bottle of milk should be discouraged;
3. Limit the frequent consumption of added sugars in foods and drinks, in particular sticky foods;
4. Limit the consumption of acidic drinks, in particular fruit juices;
5. Encourage the consumption of fluoridated tap water;
6. From 6 months of age milk and water should be offered in a cup rather than a feeding bottle;
7. Pacifiers should not be dipped in honey, jam or any other sugary substance;
8. Discourage sharing of spoons to limit the spreading of bacteria.
9. Encourage the consumption of fresh fruits and vegetables.

While government preventive health policies aim to reduce levels of sugar and fat consumption [18], current approaches in dentistry rely heavily on one-to-one dietary advice given by oral health professionals to patients [19]. Studies have reported, however, that dentists either do not give dietary advice, or if they do, it is usually of variable quality [20] or given with minimal patient interaction [21, 22]. Health education leaflets are frequently used by oral health professionals as a means of communicating dietary advice. Although the usefulness of leaflets at improving oral health outcomes is unclear, they may be useful in bridging the communication gap between dentist and patient [22] if they are designed in a clear, concise, and consistent manner, and if they adhere to accepted and current guidelines. Furthermore, many oral health professionals feel that leaflets help to reinforce information discussed with patients [22]. It is essential, therefore, that any dietary advice given as part of oral health promotion is in accordance with evidence-based nutrition guidelines, thereby strengthening the common risk factor approach to health promotion and minimising conflicting and confusing messages to the public. The aim of this study was to determine if the paediatric oral health education leaflets with a food and nutritional focus available in Australia provide messages that are clear and consistent with current Australian Dietary Guidelines (ADG) [16] and the Infant Feeding Guidelines (IFG) [17].

## Methods

### Paediatric oral health leaflets

The Australian state and territory Health Departments, oral industry partners and commercial organisations were contacted for all available oral health education leaflets pertinent to children aged 0–17 years. Hardcopy and/or online availability were determined. In the case that leaflets were made known but not readily available, the study author (AA) directly contacted the relevant publishers. Sixty leaflets were sourced.

### Qualitative content analysis

A manual qualitative analysis of the leaflets was conducted in several phases to obtain a detailed overview of the content.

#### Phase 1

All leaflets were initially screened for the presence or absence of any diet and/or nutrition-related information. Seventeen leaflets were excluded in this phase as not having any nutritional and/or dietary advice. The remaining 43 leaflets (Table 1) were from the government (n = 32), professionally-accredited associations (n = 3), independent education institutions (n = 3) and the oral health industry (n = 5). The title of the leaflet, source, publisher and year of publication were noted in this phase.

**Table 1:** Details of Australian paediatric oral health leaflets including dietary advice and/or nutritional information to prevent caries

No.	Title	Publisher^b^	Source^b^	Date	Infant diet advice^a^
1	7 tips for healthy baby teeth	Australian Dental Association	P	Unknown	Y
2	Dental care for babies and young children	Australian Dental Association	P	2007	Y
3	Oral Health for infants and toddlers	Colgate	O	2007	Y
4	Teething, Preventing tooth decay	Colgate	O	Unknown	Y
5	Oral Health for children 3-12	Colgate	O	2005	-
6	Teaching your child good brushing habits	Macleans	O	2009	Y
7	Teething	Dental Health Services Victoria	G	2010	Y
8	Tooth tips Thumb and finger sucking	Dental Health Services Victoria	G	2010	Y
9	Eat well Fact Sheet for Parents	Dental Health Services Victoria	G	2009	-
10	Drink well Fact Sheet for Parents	Dental Health Services Victoria	G	2009	-
11	Solid Kids have Healthy Teeth (Infants)	Derbarl Yerrigan Health Service	I	Unknown	Y
12	Solid Kids have Healthy Teeth (Toddlers)	Derbarl Yerrigan Health Service	I	Unknown	Y
13	Give your Child’s Teeth a Healthy Start	SA Health	G	Unknown	Y
14	Dental Information for Parents/Carers	NSW Health	G	2009	Y
15	Teach your baby to drink from a cup	NSW Health	G	2009	Y
16	NSW Messages for a Healthy Mouth	NSW Health	G	2007	Y
17	Caring for babies’ teeth	NSW Health	G	2001	Y
18	Keep your child’s teeth healthy	NSW Health	G	2007	Y
19	Tooth Smart	NSW Health	G	2011	Y
20	Good Oral Health for Children	NSW Health	G	2004	-
21	Keep smiling while you are pregnant	NSW Health	G	2009	Y
22	Healthy Mouths for Aboriginal People	NSW Health	G	2010	-
23	Healthy Smiles for Kids under 5 (Aboriginal)	NSW Health	G	2013	Y
24	Keep our Kids Smiles Strong	NSW Health	G	2011	Y
25	Sugar in Breakfast Foods	NSW Health	G	2011	-
26	Sugar in Snack Foods	NSW Health	G	2011	-
27	Tooth Smart - Grazing on Foods and Drinks	NSW Health	G	2014	-
28	Tooth Smart - Tips to eat less Sweets	NSW Health	G	2014	-
29	Tooth Smart - Tips to stop sweet drinks	NSW Health	G	2014	-
30	Tooth Smart - Goals and Rewards	NSW Health	G	2014	-
31	Tooth Smart - Reading Food and Drink Labels	NSW Health	G	2014	-
32	Tooth Smart - Stopping the Bottle (Baby)	NSW Health	G	2014	Y
33	Tooth Smart - Stopping the Bottle	NSW Health	G	2014	Y
34	Tooth Smart - Meals for children	NSW Health	G	2014	-
35	Top Dental Tips for young people	NSW Health	G	2014	-
36	Don’t rot your baby’s teeth	Queensland Health	G	Unknown	Y
37	Looking after young mouths	Queensland Health	G	Not Specified	Y
38	Caring for your child’s smile	SA Dental Service	G	Unknown	Y
39	Caring for your Child’s smile (0–6 years)	WA Dental Health Services	G	Unknown	Y
40	Thumbsucking and Dummies	WA Dental Health Services	G	2009	Y
41	Healthy eating equals healthy teeth	Australian Dental Association	P	Unknown	Y
42	Dental Care for Kids	Pacific Smiles Dental	O	2011	-
43	Decay - Whose teeth are at risk?	University of South Australia	I	Unknown	Y

^a^ “*Y*” refers to a Yes

^b^ “*P*” Professionally Accredited, “*O*” Oral Hygiene Industry, “*G*” Government, “*I*” independent, “*NSW*” New South Wales, “*SA*” South Australia, “*WA*” Western Australia

#### Phase 2

The 43 leaflets were then screened for any dietary and/or nutrition education messages or images that were confusing and/or were open to misinterpretation. If the message or the graphic was not clear, the reasons were noted.

#### Phase 3

The leaflets were screened for any infant (0–2 years) feeding advice and the responses were dichotomised. A score of 0 was given if no infant feeding advice was given. A score of 1 indicated the presence of infant feeding advice. In particular, the leaflets were screened for consistencies on the following messages:

- Any message on breastfeeding, in particular night-time breastfeeding and demand breastfeeding;
- Any advice on bottle feeding including advice on weaning and replacing sugary drinks with water; and
- Any education messages on pacifier use including advice on not dipping the dummy in sugary liquids and sharing dummies.

#### Phase 4

The leaflets were then screened for dietary and/or nutritional advice for preschool aged children (3–5 years), and school aged children (6 years to 17 years). A score of 0 was given if no dietary and/or nutritional advice was given. A score of 1 indicated the presence of dietary and/or nutritional advice. In particular, the leaflets were screened for consistencies on the following content:

- Advice to decrease the frequency and/or amount of consumption of sugary and/or acidic foods.
- Advice to restrict sugary and/or acidic foods to meal times.
- Advice on avoiding or reducing the consumption of sticky food.
- Examples of “bad” foods and/or drinks that may contain high levels of sugars, saturated fats, or salt as per the ADG and IFG guidelines.
- Advice to decrease the frequency and/or amount of consumption of sugary and/or acidic drinks.
- Advice to restrict sugary and/or acidic drinks to meal times.
- Advice on consumption of water, in particular fluoridated water.
- Advice on consumption of milk and/or other dairy products.
- Advice on consumption of fruits and vegetables.

### Analysis

STATA version 13 statistical software (Statcorp) was used for the analysis. Cronbach’s alpha was used to assess the internal consistency of leaflets targeting children aged 0–2 years, 3–5 years and 6–17 years. As a rule of thumb, an alpha > 0.9 is considered excellent, > 0.8 good, > 0.7 acceptable, > 0.6 questionable, >0.5 poor and < 0.5 unacceptable [23].

## Results

### Reliability

The internal consistency of messages was poor for leaflets targeted towards parents of infants (0–2 years) (alpha = 0.46). In comparison, alpha coefficients demonstrated that the internal consistency of the messages was acceptable for leaflets targeted towards parents of preschool aged children (2–5 years) (alpha = 0.73), and school aged children (6–17 years) (alpha = 0.75).

### Content analysis

Table 2 indicates the key dietary advice of the 43 leaflets. Twenty leaflets recommended reducing the consumption of sugary and/or acidic foods, while 23 leaflets recommended reducing the consumption of sugary and/or acidic drinks. When discussing food, sugar (n = 20) was mentioned in more leaflets than acidity (n = 4). Sugar (n = 23) was also mentioned more often than acidity (n = 8) with regard to messages around drinks. A total of 13 leaflets suggested restricting sugary and/or acidic food and/or drink consumption to meal times with ten leaflets referring specifically to food, while ten referred specifically to drinks.

**Table 2:** Dietary messages contained in leaflets^a^

No.	Sugary and/or Acidic Foods				Sugary and/or Acidic Drinks							Confusing
	Reduce	Meal Times	“Bad Foods”	Sticky Foods	Reduce	Meal Times	Water (any)	Water (fluoridated)	Milk	Flavoured Milk	Fruit Juice	Message
1	-	-	-	-	-	-	Y	Y	-	Y	Y	-
2	Y	-	Y	-	Y	Y	Y	Y	-	Y	Y	-
3	-	-	Y	-	Y	-	Y	-	-	Y	Y	-
4	-	-	-	-	Y	-	Y	Y	-	-	Y	-
5	Y	-	-	-	Y	-	Y	Y	-	-	Y	-
6	-	-	Y	-	-	-	Y	-	-	-	Y	Y
7	-	-	Y	-	-	-	Y	-	Y	-	-	-
8	-	Y	Y	Y	-	-	Y	Y	Y	-	Y	-
9	Y	Y	Y	Y	Y	Y	Y	-	Y	-	Y	-
10	-	-	-	-	Y	-	Y	Y	Y	-	Y	Y
11	-	-	Y	-	-	-	Y	-	Y	-	-	Y
12	Y	Y	Y	-	-	Y	Y	-	Y	-	-	-
13	-	-	-	-	-	-	Y	Y	Y	Y	Y	-
14	-	-	Y	Y	-	-	Y	Y	Y	-	Y	Y
15	-	-	-	-	Y	-	Y	-	Y	-	Y	-
16	Y	-	Y	Y	Y	-	Y	Y	Y	-	Y	-
17	-	Y	-	-	Y	Y	Y	-	-	-	Y	-
18	-	-	-	-	-	-	Y	Y	Y	-	-	-
19	-	Y	Y	Y	-	Y	Y	Y	Y	Y	-	Y
20	Y	-	Y	-	Y	-	Y	Y	Y	Y	Y	-
21	Y	Y	Y	-	Y	-	Y	-	Y	-	Y	Y
22	Y	-	Y	-	Y	-	Y	Y	-	-	-	-
23	Y	-	Y	Y	Y	-	Y	Y	Y	-	Y	-
24	-	-	-	-	-	-	Y	-	Y	-	-	-
25	-	Y	Y	-	-	-	-	-	-	-	-	-
26	-	-	Y	-	-	-	Y	-	-	-	-	-
27	Y	-	-	-	Y	-	-	-	-	-	-	-
28	Y	-	Y	-	-	-	-	-	-	-	-	-
29	-	-	-	-	Y	Y	Y	-	-	Y	Y	-
30	Y	-	-	-	-	-	-	-	Y	-	-	-
31	Y	-	-	-	Y	-	-	-	-	-	-	-
32	-	-	-	-	-	-	Y	-	Y	-	-	-
33	-	-	-	-	-	-	Y	-	Y	-	-	-
34	Y	-	Y	-	Y	-	-	-	Y	Y	-	-
35	Y	-	-	Y	Y	Y	Y	Y	Y	-	-	-
36	-	Y	-	-	Y	Y	-	-	-	-	-	-
37	Y	Y	Y	-	Y	Y	Y	-	Y	-	Y	Y
38	Y	-	-	-	Y	-	Y	Y	-	-	Y	-
39	Y	-	-	Y	-	-	Y	-	-	-	Y	Y
40	-	-	-	-	-	-	-	-	-	-	-	-
41	Y	-	Y	-	Y	-	Y	Y	Y	-	Y	Y
42	Y	-	-	-	Y	-	Y	Y	-	-	Y	-
43	-	Y	-	-	-	Y	Y	Y	-	-	Y	-

^a^ “Y” refers to a Yes

The majority of leaflets (n = 35) recommended water to drink while 23 leaflets recommended milk. Nineteen leaflets specifically advised drinking fluoridated or tap water. Eight leaflets made a distinction that flavoured milk was not beneficial. Just over half of the leaflets (n = 24) identified fruit juice as a potential contributor to tooth decay.

Over half of the leaflets specified examples of unhealthy drinks (n = 25), while only 19 leaflets gave specific examples of unhealthy foods. Five leaflets recommended completely removing sweets from the diet and only eight leaflets advised avoidance of “sticky” foods.

### Incomplete messages

Four leaflets provided general information about oral health and nutrition, but did not provide any specific recommendations or guidance. For example, leaflets 1 and 3 described foods and/or drinks that could potentially increase the risk of early childhood caries, but did not suggest any alternatives that could be placed in the baby bottle or clearly state the way foods/and or drinks should be consumed.

### Confusing messages

Nine leaflets contained potentially confusing messages. Some examples are listed in Table 3.

**Table 3:** Examples of unclear and confusing information in leaflets

No.	Leaflet content	Potential for confusion/misinterpretation
6	“Foods that are high in sugar (such as lollies and soft drinks) or food acids (such as some fruit juices) can be bad for teeth as they can lead to cavities or loss of tooth enamel.”	States ‘some’ fruit juices can be bad. Does not expand further on which types can be good or which can be bad.
12	Images to demonstrate food and drinks to “avoid” without any written message	Use of images without clear labelling to identify what they are supposed to represent could lead to misinterpretation.
19	“Fruit Drinks (with added sugar)” (Under title: “Stop”)	Fruit Drinks is an ambiguous term, as it could refer to juice, cordial, or a fruit-flavoured beverage. Also suggests drinks such as fruit juice with no added sugar are okay.
39	“This will help prevent ‘Early Childhood Caries’ which is decay caused by frequent, prolonged use of a bottle containing sweet liquids such as milk, juice or cordial.” AND “Water (and milk in moderation) is the best thirst quencher.”	Contradicts by linking milk to causing Early Childhood Caries, then later states that milk in moderation is the best thirst quencher.
41	“Offer a diet high in fresh fruits and vegetables, wholegrain cereals, lean meats and dairy products.” AND “Many healthy foods (such as fruit) contain high amounts of sugar.”	Contradicts by recommending fruit in diet, then later states that it is high in sugar.

### Snack recommendations

Thirty-three leaflets advised to eat a “healthy diet”. Seven of these leaflets however ambiguously referred to “healthy foods” or a “nutritious diet” without further examples. Of those leaflets that provided healthy snack recommendations (n = 25), all indicated fruit and vegetables, while the majority also suggested cheese and dairy products (Table 4).

**Table 4:** Leaflet content in relation to snack recommendations ^a^

No.	Fruits & Vegetables	Cheese	Dairy
1	-	-	-
2	Y	-	Y
3	Y	-	-
4	-	-	-
5	Y	-	Y
6	Y	Y	Y
7	Y	-	Y
8	Y	Y	Y
9	Y	Y	Y
10	Y	-	-
11	-	-	-
12	Y	Y	-
13	-	-	-
14	Y	Y	-
15	Y	Y	Y
16	Y	Y	-
17	-	-	-
18	-	-	-
19	Y	Y	Y
20	Y	-	Y
21	Y	-	Y
22	Y	Y	Y
23	Y	Y	Y
24	-	-	-
25	-	-	-
26	Y	Y	-
27	Y	Y	Y
28	Y	-	-
29	-	-	-
30	Y	-	Y
31	-	-	-
32	-	-	-
33	-	-	-
34	Y	Y	Y
35	Y	Y	Y
36	-	-	-
37	Y	Y	Y
38	-	-	-
39	-	-	-
40	-	-	-
41	Y	Y	Y
42	-	-	-
43	-	-	-

^a^ “Y” refers to a Yes (contained in leaflet) ‘-‘refers to a ‘No’ (not contained in leaflet)

### Feeding and pacifier advice for 0–2 years

Table 5 details the content of 28 leaflets in the sample that provided advice in relation to feeding and pacifier use for 0–2 year olds (infants). Although 25 of the 28 leaflets advised limiting bottle feeding in general, only 17 advised against night-time bottle feeding. Eleven leaflets recommended placement of water in the bottle for prolonged feeding, and seven gave specific examples of fluids that should not be placed in the bottle. Although ten leaflets encouraged breastfeeding, none specifically promoted the optimal duration of exclusive and continued breastfeeding of 6 and 12 months or more, respectively. Nearly half of these leaflets (n = 14) suggested introducing a cup at age 6 months and only four leaflets suggested to start solid foods at age six months. Ten leaflets gave advice on the use of a pacifier, all of which advised not to dip the pacifier in sugary products such as honey or jam.

**Table 5:** Content analysis of infant feeding and pacifier advice in leaflets aimed at early childhood (aged 0–2 years)^a, c^

No.	Bottle Feeding	Water in bottle	No sugary fluids in bottle^b^	Overnight feeding	Breast milk	Pacifier advice	Dipping pacifier in sugar
1	Y	-	Y	Y	-	-	-
2	Y	Y	-	-	-	Y	Y
3	Y	Y	Y	-	-	Y	Y
4	Y	Y	-	-	-	Y	Y
6	Y	Y	-	-	-	-	-
7	Y	-	-	Y	Y	Y	Y
8	Y	-	-	-	-	-	-
11	Y	Y	-	Y	Y	Y	Y
12	Y	Y	-	Y	-	Y	Y
13	Y	-	-	Y	Y	Y	Y
14	Y	-	Y	Y	Y	-	-
15	Y	-	Y	Y	Y	-	-
16	Y	Y	-	-	Y	-	-
17	Y	Y	Y	Y	-	-	-
18	Y	-	-	Y	Y	-	-
19	Y	-	-	-	-	-	-
21	Y	-	-	Y	Y	-	-
23	Y	-	Y	Y	-	-	-
24	Y	-	-	Y	-	-	-
32	Y	Y	-	Y	-	-	-
33	Y	-	-	-	-	-	-
36	Y	-	-	Y	Y	-	-
37	Y	-	-	Y	Y	Y	Y
38	-	-	-	-	-	-	-
39	Y	Y	-	Y	-	Y	Y
40	-	-	-	-	-	Y	Y
41	-	-	-	-	-	-	-
43	Y	Y	Y	Y	-	-	-

^a^ From a total of 24 infant-targeted leaflets

^b^ Sugary fluids include milk, juice & cordial

^c^ “Y” refers to a Yes

### Use of images

Six leaflets used relevant images to accompany the message being delivered, however some of these did not have any or appropriate words accompanying the image (Table 3).

## Discussion

The findings of this study indicate that the dietary advice and nutritional information in leaflets aimed at preventing tooth decay in children in Australia does not always align with the national dietary and infant feeding guidelines. In addition, many of the important items of information were inconsistently included across leaflets, with each individual leaflet lacking at least one key item of information and few leaflets containing the majority of or all key items. Further, some dietary messages to prevent dental decay were unclear and hence could be subject to misinterpretation by individuals.

The majority of the leaflets did not contain any recommendations specific to the amount and timing of sugary and/or acidic food consumption, i.e. key information specific to dietary prevention of caries. The ADGs [16] emphasise established evidence with respect to the amount, timing and frequency of consumption of these food types as important in the prevention of tooth decay. [24]. It is generally recommended to restrict sugar to meal times, so the tooth enamel is given an opportunity to remineralise and return to a neutral pH [25]. In contrast, frequent snacking on sugary foods and drinks between main meals allows for continuous acid attack on tooth surfaces [25]. Banning sugary foods and drinks is a practice parents could be advised to adopt however more realistically parents should be advised to reduce the frequency of consumption of sugary foods by their children and restrict the consumption of sugary foods by their children to meal times only.

‘Acidity’ was mentioned less frequently than ‘sugar’ in the education leaflets. Although sugar consumption has a well-known association with tooth decay [6, 7], acidity also has relevant dental implications in the form of tooth erosion leading to possible complications such as severe tooth surface loss, tooth sensitivity, poor aesthetics, and dental abscess [26]. With increased marketing of sugar-free soft drinks and isotonic sports drinks, parents may be misled to believe that these make for better options for their children compared to their sugary counterparts. While this may be true with regards to a decreased risk for dental caries and obesity, these sugar-free beverages have negative dental consequences such as tooth erosion. It is important that more awareness is raised regarding the acidic content of sugar-free drinks and isotonic sports drinks so that parents can make more informed decisions.

In accordance with the ADG [16] and IFG [17], water consumption was advocated in the majority of leaflets. Tap/public water, which contains fluoride in most areas of Australia, was advised in more than half of the leaflets. The distinction between fluoridated tap water and non-public water such as bottled water, however, was not explicitly stated in all leaflets. Consumption of fluoridated tap water should be more strongly advocated as there is scientific evidence that the consumption of non-public water is positively associated with dental decay [27, 28]. Further, while the majority of Australian states and territories are fluoridated [29], water in some rural areas remains non-fluoridated and this is a concern and therefore benefits of water instead of other drinks should be advocated to these populations [30].

Daily consumption of fruits and vegetables as recommended in the ADG [16], was consistently recommended by all leaflets that suggested healthy food examples. Aside from their commonly-known nutritional benefits, oral health benefits have been found in certain fruits. It has been reported that consumption of fibrous fruit after meals is potentially preventive against tooth decay due to the cleansing action and increased salivary flow [13]. Conversely, Arora et al. [31] demonstrated that frequent exposure to fruits was positively associated with dental decay, although causal evidence to support this association is lacking.

The ADG [16] mention avoiding “sticky” food, with a particular reference to dried fruit. Only one in five of the leaflets identified sticky foods as a problem with implications for dental caries. The longitudinal Vipeholm study [24] reported that sticky foods consumed between meals increased the risk of decay due to their sticky texture, allowing for greater adherence to the tooth surface leading to longer exposure time to the tooth enamel. This ultimately means that there is slower clearance of the sticky foods, resulting in a continual acid attack without chance of tooth remineralisation [32]. Therefore it is important to highlight the importance of food textures, primarily ‘stickiness’, in parent education regarding prevention of dental decay in their children.

The ADG [16] and IFG [17] suggest that milk and dairy are excellent sources of calcium and should be consumed after 12 months of age. However, relatively few leaflets recommended milk and/or cheese as healthy snack options. Apart from general health benefits [33], both these dairy products have proven positive effects in reducing the negative impact of metabolic acids on teeth and restoring lost tooth enamel during the remineralisation process [34]. Although milk is a concern for early childhood caries due to pooling when fed through a bottle [6], it has health benefits [35] and anti-cariogenic effects when consumed appropriately. The dental benefits of cheese are less well-known and are due to several mechanisms. Herod [34] stated that chewing cheese leads to an increased stimulation of saliva which results in increased buffering of acids found in dental plaque and also delivers calcium and phosphate which enhances remineralisation [36].

The most prevalent bottle feeding message found in these leaflets advised not to leave an infant with a bottle of milk overnight, which is consistent with relevant message advocated in the ADG [16] and IFG [17]. Although some parents may find it difficult to put their child to sleep without the comfort of a bottle, it is healthier to allow a child to drink milk before bedtime and then follow this with toothbrushing. Only seven of the 43 leaflets discouraged filling bottles with other fluids such as fruit juice or cordial, rather than water. The lack of attention to this preventive health recommendation in paediatric dental health leaflets is a special cause for concern and requires remedying.

Breastfeeding was advocated in ten leaflets, none of which explicitly advised exclusive breastfeeding until 6 months of age as recommended by the ADG [16] and IFG [17]. A review by Ribeiro & Ribeiro [37] found that breast milk had less potential to cause tooth decay than cow’s milk and infant formula. Other studies have shown that breastfeeding transfers antibodies responsible for hindering bacterial growth, and the lactoferrin in breast milk has a bactericidal effect on cariogenic bacteria [38].

The practice of dipping a pacifier in sugary products to help put infants to sleep was discouraged in only some leaflets targeted at parents of infants. Bedtime feeding of sugary substances either in a bottle or as a coating on a pacifier not only has implications related to early childhood decay, but the increased caloric intake can impact negatively on general health by predisposing to obesity [39]. This message should be a core component of relevant leaflets.

Just over half (n = 24) of leaflets accurately identified fruit juice negatively with respect to dental caries. Fruit juices are generally acidic and contain free sugars – both of which have detrimental effects on teeth [7]. Conversely, fresh fruit is often considered a healthy food and is included as a healthy snack recommendation in the ADG [16]. It is important for parents to be educated on the differences between fruit juice and fresh fruit, particularly with regard to the free sugar and acid content in respect of dental health [7, 31, 40].

In this study, more leaflets provided examples of recommended “good” foods and drinks, rather than providing examples of “bad” foods and drinks to avoid. The significance of the use of specific language in conveying health-related messages has been highlighted in the past [41]. More recently, it was reported in an Australian study that mothers of young children found the advice on bottle feeding confusing due to the use of language [42]. This is consistent with the idea that negative framing of messages, such as those encouraging people to eat a healthy diet, promotes avoidance behaviour while positive framing facilitates performance of prevention behaviours [43]. The effect of negative versus positive framing of prevention messages in dental health leaflets deserves research attention.

Pictures accompanied by simple phrases and captions have been shown to be beneficial in conveying health messages to patients as they improve comprehension and recall [44]. Research in experimental psychology and marketing highlights that humans have a cognitive preference for picture-based rather than text-based information: the so-called “picture-superiority effect” [45]. On the other hand, poorly captioned images and the use of ambiguous terms can detract from the intended message. There were some examples of ambiguous images and supporting text in the leaflets in this study. Leaflet 12, for example, had an image recommending avoidance of “cool drinks”, an Australian expression sometimes used to describe soft drinks or sugar-sweetened beverages, but provided no further clarification. As a result, parents may interpret this image and recommendation incorrectly and think that all cold drinks, including water and milk, are unhealthy. Additionally the message implies a false idea that any type of “warm drink” is acceptable, which may not be the case; it has been reported that acidic drinks at room temperature increase the risk for dental erosion. The need for clearer health education images and accompanying and concurrent messages is therefore warranted [46].

Although leaflets were sourced from most state and territory health departments and a variety of other organisations, the authors acknowledge that not all leaflets may have been located hence that the findings of this study may not be representative of all dental education leaflets in Australia.

## Conclusions

There were some discordance between the informational content across paediatric dental health promotion leaflets and dietary and infant feeding guidelines in Australia. In addition, some dietary and infant feeding messages and imagery lacked clarity. Inconsistencies in similar pieces of information also occurred across leaflets; and few leaflets contained most or all of the pertinent dietary and infant feeding information messages to help prevent dental decay and erosion in children. Government Health Departments and other relevant agencies should ensure that advisory messages regarding diet and infant feeding, are clear, complete and consistent across all dental educational leaflets in Australia.

The core diet-related messages that should be included in ideal paediatric oral health leaflets are:

1. Infants should be exclusively breastfed until around 6 months of age followed by the introduction of solid foods; and breastfeeding should be continued until 12 months of age and beyond;
2. If infants are not breastfed, infant formulas should be used as an alternative to breast milk until 12 months of age;
3. Infants should not go to bed with a bottle of milk, soft drink or fruit juice;
4. The frequent consumption of added sugars in foods and drinks, and in particular sticky foods with meals should be limited;
5. The consumption of acidic drinks, in particular fruit juices and carbonated beverages, should be limited;
6. Drink fluoridated tap water;
7. Introduce a sipper cup from age 6 months;
8. Pacifiers should not be dipped in honey, jam or any other sugary substance;
9. Do not share spoons or other eating utensils such as forks and chopsticks;
10. Eat two serves of fruit and five serves of vegetables daily.

## Abbreviations

ADG: Australian dietary guidelines
IFG: Infant feeding guidelines
NHMRC: National health and medical research council
NSW: New South Wales
SA: South Australia
UNSW: University of New South Wales
WA: Western Australia
WHO: World Health Organisation
